# Prospective Pathways Among Rumination, Depression, and Insomnia in Youth

**DOI:** 10.1007/s10802-025-01406-x

**Published:** 2026-01-22

**Authors:** Brooklynn Bailey, Allison K. Wilkerson, Melanie A. Stearns, Rachel E. Siciliano, Carla Kmett Danielson

**Affiliations:** 1 Department of Psychiatry and Behavioral Sciences, Medical University of South Carolina, Charleston, SC USA; 2 College of Nursing, University of South Florida, Tampa, FL USA

**Keywords:** Insomnia, Sleep, Depression, Rumination, Longitudinal, Mediation

## Abstract

A growing body of research suggests sleep disturbances and depressive symptoms do not just co-occur but influence each other over time. While emerging evidence supports a bidirectional relationship, developmental pathways between sleep problems and depression remain understudied in youth. Rumination may serve as a potential modifiable cognitive mechanism linking these conditions. However, rumination’s role in these developmental pathways remains largely unexplored, particularly across childhood and adolescence. This study employed a cross-lagged panel model (CLPM) to investigate prospective associations among sleep, depression, and rumination and test mediation effects. Participants were 364 youth aged 8 to 16 participating in three yearly assessments. A distinct pattern emerged in which rumination predicted later depression, and depression predicted later insomnia. Insomnia did not significantly predict later depression and was not associated with rumination. Examination of indirect effects identified support for a mediational pathway from rumination to insomnia through depression. Although prior research has framed early insomnia symptoms as a risk factor for later depression, the present investigation highlights an alternative developmental pathway. Results suggest that depressive rumination contributes to later depression which in turn contributes to later insomnia among youth. These findings may inform prevention and early intervention efforts by identifying rumination as a potential target to mitigate risk for depression and insomnia.

## Introduction

The early onset of sleep and mood problems confers risk for more severe and persistent difficulties across the lifespan (Fernandez-Mendoza et al., 2022; Fernando et al., 2011; Korczak & Goldstein, 2009). This underscores the importance of understanding the developmental pathways and modifiable mechanisms which contribute to the emergence of sleep disturbances and depression in youth. A connection between sleep and depression has been demonstrated in children and adolescents (Asarnow & Mirchandaney, 2021; Lovato & Gradisar, 2014; Marino et al., 2021) as well as adults (Baglioni et al., 2011; Zhai et al., 2015). Of the forms of sleep disturbance, insomnia- characterized as difficulty falling and staying asleep- emerges as a central focus of study because it is the most prevalent and most strongly linked to depression (Franzen & Buysse, 2008; Zhang et al., 2022). A growing body of research suggests sleep disturbances, including insomnia, and depressive symptoms do not just co-occur but actively influence each other over time (Alvaro et al., 2013; Marino et al., 2022; Roberts & Duong, 2014; Sivertsen et al., 2021). While there is emerging evidence for a bidirectional relationship, the developmental pathways among insomnia and depression have been minimally studied in youth (Sivertsen et al., 2021). Recent research suggests that rumination may serve as a mediator in this relationship, acting as a modifiable cognitive mechanism through which insomnia and depressive symptoms interact (Li et al., 2024; Richardson et al., 2025). However, the role of rumination in these potentially bidirectional associations remains largely unexplored, particularly across childhood and adolescence. Given the importance of prevention and early intervention efforts, understanding the developmental sequence and mechanisms, including rumination, that drive the insomnia-depression link in youth is paramount.

### The Sleep and Depression Relationship

While closely linked, insomnia is recognized as a distinct clinical condition from depression which often persists after the remission of other depressive symptoms (Franzen & Buysse, 2008). The rate of comorbid insomnia and depression in school-aged children and adolescents is high (Emslie et al., 2012; Liu et al., 2007; Roberts & Duong, 2014) and appears to strengthen with age (Gregory & O’Connor, 2002; Johnson et al., 2000). Clarifying the sequence in which symptoms emerge is crucial for understanding the development and progression of these comorbid difficulties. Insomnia has long been recognized as a risk factor for later depression (Franzen & Buysse, 2008). Indeed, prior systematic reviews and meta-analyses in children and adolescents found evidence for a unidirectional relationship where sleep difficulties (including insomnia) predicted later depression, with little-to-no evidence that depression predicted later sleep difficulties in youth (Alvaro et al., 2013; Lovato & Gradisar, 2014). In contrast, these same studies identified reciprocal relationships between depression and insomnia among adults.

Nevertheless, more recent research has detected prospective, bidirectional relationships between sleep disturbances and depression in youth, in line with findings from adult samples. Bidirectional relationships have been identified across the large developmental period of early toddlerhood to childhood (Sivertsen et al., 2021) and across year-to-year assessments in childhood (Marino et al., 2022). Still, recent studies continue to cast doubt on whether this bidirectional relationship extends to adolescence (Marino et al., 2022; Quach et al., 2018; Richardson et al., 2025). Further research is needed examining the directionality of depression and insomnia development across youth and the mechanisms underlying this link.

### The Role of Rumination as a Potential Mediator

The mechanisms connecting insomnia and depression are likely multifactorial, including both genetic and environmental processes (Sivertsen et al., 2021). Among these, rumination- passive, repetitive thinking on the causes, experience, and consequences of one’s distress- may be may be of particular clinical relevance given its modifiability (Nolen-Hoeksema et al., 2008; Watkins & Roberts, 2020). Rumination is a well-established risk factor for depression onset (Nolen-Hoeksema et al., 2008; Watkins & Roberts, 2020), and prominent theories of insomnia also implicate rumination in its development and maintenance (Blake et al., 2018; Tang et al., 2023). Indeed, a systematic review and meta-analysis found rumination is associated with sleep problems, including poorer sleep quality, shorter sleep duration, and longer sleep onset latency (Clancy et al., 2020). Most research on rumination has focused on ruminative tendencies in response to low mood, or depressive rumination (Nolen-Hoeksema et al., 2008), though measures have been developed to capture rumination occurring specifically before bed or around sleep-related distress, as well as general, symptom-independent rumination (Clancy et al., 2020).

It is theorized that sleep problems may contribute to the development of internalizing disorders, such as depression, by increasing susceptibility to rumination, potentially through the disruption of brain networks during the sensitive developmental transition from childhood to adolescence (Akbar et al., 2022). Sleep patterns change for adolescents due to a shift in circadian rhythm where they experience delayed bed and wake times (Gradisar et al., 2011). Unfortunately, earlier school start times, in combination with shifted bedtimes, result in more than 50% of adolescents getting less than the recommended amount of sleep (8–10 h) (Meltzer et al., 2021). In adolescents, sleep problems (e.g., less total sleep, later bedtimes) have been associated with cortical thinning in areas of the default mode network, which is connected to mind wandering and thinking about the past and future characteristic of rumination (Jalbrzikowski et al., 2021). This was not observed in young adults, further suggesting that the transition to adolescence may be a critical developmental period for sleep which may affect tendencies to ruminate and experience depression.

Although the bulk of studies have examined rumination and insomnia in adolescents, these concepts are increasingly recognized as relevant and clinically significant in childhood, with growing evidence linking rumination and sleep disruptions to early-onset emotional and behavioral difficulties. Rumination has been observed in children as young as 7 and is associated with heightened risk for internalizing problems, including anxiety and depression (Harmon et al., 2019). Similarly, insomnia affects approximately 20–30% of school-aged children and is linked to impairments in emotion regulation, attention, academic performance, and family functioning (Owens & Palermo, 2008). Importantly, these processes often interact: children who ruminate at bedtime may experience heightened physiological arousal and difficulty initiating sleep, contributing to a cycle of sleep disruption and mood dysregulation (Palmer & Alfano, 2017).

Similarly, depression in childhood, though less prevalent than in adolescence, affects roughly 2–3% of children and carries significant developmental consequences (Psychogiou et al., 2024). Early-onset depression is associated with increased risk for recurrent episodes, academic failure, social withdrawal, and suicidality in adolescence and adulthood (Thapar et al., 2012). Given that both rumination and insomnia are implicated in the onset and maintenance of depressive symptoms, their presence in childhood warrants focused attention in developmental psychopathology research. Indeed, recent empirical research also suggests rumination may be a key mediator in the relationship between sleep and depression. Among adolescents aged 12 to 16 (*N* = 264), Li and colleagues (Li et al., 2024) found symptom-independent rumination partially mediated the relationship between insomnia and depressive symptoms in a cross-sectional analysis. This mediating role was also identified for baseline rumination when examining post-intervention depressive symptoms among a subset of their sample participating in an active control condition for insomnia (Li et al., 2024). Interestingly, unhelpful beliefs about sleep did not mediate these relationships, suggesting this role may be specific to the ruminative cognitive process versus cognitive content or cognitive factors broadly.

As the Li et al. (Li et al., 2024) study did not examine the effect of depression on later insomnia, it is unclear whether rumination would also mediate this relationship in youth. Although, two studies among adults provide initial evidence it might. In 165 young adults, the effect of depressed mood on poorer sleep quality two months later was partially mediated by depressive rumination at follow-up (Slavish & Graham-Engeland, 2015). In a primarily adult sample with insomnia (*N* = 924), the effect of depressive symptoms on later insomnia was mediated by only rumination, catastrophizing, and emotional reactivity out of 17 potential mediators tested over a period of three months (Tsui & Chan, 2024). This mediational pattern has also been identified cross-sectionally in a large sample (*N* = 1240) of adults (Wang et al., 2023). Moreover, this work was expanded by Richardson and colleagues (Richardson et al., 2025) who examined several potential mediational pathways for sleep, depression, and pre-sleep and general rumination among 528 adolescents beginning at age 11 and completing yearly assessments through age 16 (Richardson et al., 2025). The strongest support was found for rumination (both pre-sleep and general) partially mediating the effect of sleep on later depression over time. However, results suggest other mediational pathways may also be at play, such as sleep partially mediating the effect of rumination on later depression, which was also significant in their models but smaller in effect.

Taken together, these studies suggest rumination may play a key role in the development of sleep disturbances and depression in adolescent and adult samples. To date, no known studies have explored the role of rumination in the development of sleep problems and depression from childhood through adolescence.

### Study Aims and Hypotheses

The purpose of the present study was to assess prospective, reciprocal relationships among insomnia, depression, and rumination in youth and to clarify rumination’s role in these developmental pathways. These aims were examined in a community sample of youth aged 8 to 16 participating in three yearly assessments. To date, most studies have assessed these variables concurrently or with overlapping timeframes, limiting the ability to draw causal influences. This study will address the need for longitudinal designs, leveraging a three-variable cross-lagged panel model (CLPM) to disentangle directional effects and test mediational pathways.

It was hypothesized that insomnia and depressive symptoms would exhibit reciprocal relationships over time. Rumination at year 1 was hypothesized to mediate the effect of early insomnia on later depression as well as the effect of early depression on later insomnia. All developmental sequences were tested to allow for exploratory examination of alternative pathways.

Depression and rumination severity were child-reported, while insomnia severity was reported by caregivers. Caregivers can reliably report on children’s behaviors surrounding sleep onset and daytime impairment, but often struggle to accurately assess their child’s overall sleep patterns (McDowall et al., 2017). To address this limitation, a child-reported measure of overall sleep/wake problems was included as a secondary outcome.

## Methods

Data were drawn from a community sample of 364 youth as a part of a NIMH-funded longitudinal study of risk and resilience factors and mental health. Eligibility criteria included youth: (1) ages seven to 16 years of age; (2) in the third, sixth, or ninth grade; (3) with a caregiver willing to participate; (4) ability to speak and write in English; (5) no history of psychosis or developmental delay interfering with study procedure completion. The sample spanned developmental cohorts with *n* = 117 in 3rd, *n* = 128 in 6th, and *n* = 119 in 9th grade cohorts (*M*age = 11.49, *SD* = 2.47). The sample was 49.5% female, and participants identified as White (52.2%), Black/African American (38.7%), and Hispanic/Latino (8.8%).

### Procedures

Youth were recruited from the community via branding materials, social media posts, school events, community fairs and festivals, community and pediatric primary care clinic flyers, recruitment letters from schools, local newspapers and magazines, and a study-dedicated website. Eligibility was determined by phone screens completed by a study team member. Eligible participants were invited to complete the multi-wave, two-year study, which included a baseline laboratory visit, six-month phone call, 12-month laboratory visit, 18-month phone call, and 24-month follow-up laboratory visit. Data presented here are drawn from the larger study and include youth and caregiver surveys gathered from the baseline, 12-, and 24-month visits. All study procedures were approved by the Medical University of South Carolina institutional review board. Before enrolling in the study, all youth participants and their caregivers provided written assent and written informed consent, respectively, and were compensated for their participation.

### Measures

#### Demographics

Caregivers reported demographic information, including their child’s age, sex/gender (male, female, other [e.g., transgender, undecided, ambiguous, gender fluid, agender, etc.]), race, and ethnicity. Analyses utilized grade cohort (3rd, 6th, 9th) and sex as covariates. Sex was coded as female (1) and male (0), as all caregivers reported their child as male or female.

#### Insomnia

Sleep disturbances were measured using the caregiver-report measure: the Sleep Disturbance Scale for Children (SDSC) (Bruni et al., 1996). The SDSC includes several subscales characterizing children’s sleep over the past six months. To examine insomnia, this study utilized the 7-item disorders of initiating and maintaining sleep subscale as the primary sleep variable. This subscale includes items on sleep duration, sleep onset latency, awakenings after sleep onset, and anxiety and reluctancy around sleep. Scores range from 7 to 35, with higher scores reflecting greater sleep difficulty. The SDSC total score has demonstrated good internal consistency in clinical and nonclinical samples of children and adolescents (Cronbach’s α = 0.71-0.79), and the disorders of initiating and maintaining sleep subscale has been shown to distinguish youth with and without sleep disorders (Bruni et al., 1996). Reliability in the current sample was adequate (Cronbach’s α = 0.70), indicating acceptable internal consistency among the scale items.

####  Sleep Difficulties

Youth participants completed the 15-item Sleep Habits Survey (SHS) about the frequency of sleep difficulties in the past two weeks (Wolfson & Carskadon, 1998). While the SHS is not an insomnia-specific measure, the 10-item sleep/wake subscale captures sleep difficulties characteristic of insomnia, including difficulty falling asleep at night and sleepiness during the day. This subscale was used to capture youth-reported sleep difficulties in secondary analyses. Scores range from 10 to 50, with higher scores indicating more frequent sleep difficulties. The SHS has excellent validity and reliability compared to actigraphy and sleep diaries (Wolfson et al., 2003). While developed for adolescents, this measure has also been used in research with younger children (El-Sheikh et al., 2013). The scale demonstrated good internal consistency in the present study (Cronbach’s α = 0.84).

#### Depression

Depressive symptoms were assessed using the 27-item Children’s Depression Inventory (CDI) (Kovacs, 1992), one of the most widely used youth self-report measures of depressive symptoms. The CDI measures affective, cognitive, and behavioral symptoms of depression in children and adolescents experienced in the past two weeks. Total scores range from 0 to 54, with higher scores indicating greater severity. The CDI is highly correlated with other depression measures in youth and exhibits high internal consistency in clinical and community samples (Masip et al., 2010; Reynolds, 1998). Primary analyses utilized the CDI total score excluding sleep and tiredness items to address potential construct overlap with sleep measures. The scale demonstrated good internal consistency in the present study (all items: Cronbach’s α = 0.88; excluding sleep items: Cronbach’s α = 0.88).

#### Rumination

After completing the CDI, youth participants completed the Children’s Response Style Questionnaire (CRSQ) which includes a 13-item subscale of ruminative responses to sad mood in addition to subscales assessing use of distraction and problem-solving (Abela et al., 2004). As part of the rumination subscale, youth participants reported on how often they typically engage in ruminative cognitive processes when feeling sad, such as brooding on negative content. The CRSQ does not reference a specific time frame but instead asks youth to consider their general tendencies. Items were coded such that scores range from 0 to 39, with higher scores signifying higher ruminative tendencies in response to depressed mood. The CRSQ rumination subscale has shown good internal consistency (Cronbach’s α = 0.82) in youth samples (Abela et al., 2004, 2007). Reliability in the present study was excellent for the rumination subscale (Cronbach’s α = 0.91), indicating strong internal consistency.

### Analytic Strategy

The present study employed a cross-lagged panel model (CLPM), implemented in R 4.3.0 with the package lavaan. This modelling approach confers key methodological advantages. CLPMs control for the influence of prior levels of a variable on later levels (i.e., autoregressive effects) and simultaneously tests for reciprocal relationships within one model (i.e., cross-lagged effects). The primary model included observed measures of insomnia (SDSC subscale), depression (modified CDI), and rumination (CRSQ subscale) at three time points. In a secondary model, the observed variable of child-report sleep difficulties (SHS) was examined in lieu of caregiver-reported insomnia. This three-variable CLPM design enabled the examination of longitudinal mediation by allowing each variable to serve as a predictor (baseline), mediator (year 1), and outcome (year 2). Indirect effects (i.e., path a × b) were estimated for all six possible longitudinal mediation pathways.

The lavaan function used Full Information Maximum Likelihood, a robust method for handling missingness that makes use of all available data under the missing-at-random assumption. To determine the optimal model structure, three nested models were compared: (1) an unconstrained model; (2) a model with autoregressive paths constrained to be equal across time points; (3) a model with both autoregressive and cross-lagged paths constrained to be equal. The best fitting model was selected based on lowest Akaike Information Criterion (AIC), Bayesian Information Criterion (BIC), and Root Mean Square Error of Approximation (RMSEA) indices. Next, the covariates of sex (0 male, 1 female) and grade (3, 6, 9) were included as predictors of each observed variable, with effects constrained to be equal across time points.

Absolute model fit was judged based on the following recommendations for structural equations models: Comparative Fit Index (CFI) and Tucker-Lewis Index (TLI) greater than or close to 0.95; RMSEA less than or close to 0.06 (Hu & Bentler, 1999). Standardized cross-lagged effect sizes were interpreted based on benchmarks identified by Orth et al. (2024): 0.03 (small effect), 0.07 (medium effect), and 0.12 (large effect). Consistent with standard reporting practices, conventional *p*-values are evaluated against 0.05 (significant) and 0.08 (trend-level) thresholds. Since indirect effects often have non-normal sampling distributions, 

## Results

Means, standard deviations, and correlations among insomnia, depression, and rumination scores are reported in Table 1. Insomnia scores in the current sample were approximately one standard deviation higher than those observed in the non-sleep-disordered control group from the original validation study (Bruni et al., 1996) but remained below the levels reported for clinically sleep-disordered youth, falling roughly 0.7 standard deviations below that group’s mean. Meanwhile, depression means and standard deviations aligned with typically reported levels in non-clinical samples (Reynolds, 1998). About 49% of the sample screened for disordered sleep and 14% screened for clinical depression at any point in the study based on SDSC and CDI cutoffs (Kovacs, 1992; Masip et al., 2010). Depression and rumination scores were strongly correlated. In contrast, associations between rumination and the sleep variables were non-significant or, when significant, weak. Depression was weakly correlated with caregiver-reported insomnia, with a stronger relationship with youth-reported sleep difficulties.

**Table 1:** Descriptive statistics and correlations for variables at baseline (1), year 1 (2), and year 2 (3)

	IN1	IN2	IN3	SLP1	SLP2	SLP3	DEP1	DEP2	DEP3	mDEP1	mDEP2	mDEP3	RU1	RU2	RU3
IN1	-														
IN2	0.60***	-													
IN3	0.54***	0.62***	-												
SLP1	0.31***	0.23***	0.29***	-											
SLP2	0.22***	0.14*	0.23***	0.44***	-										
SLP3	0.15**	0.15*	0.27***	0.43***	0.58***	-									
DEP1	0.20***	0.29***	0.22***	0.42***	0.28***	0.30***	-								
DEP2	0.14*	0.27***	0.27***	0.25***	0.40***	0.36***	0.63***	-							
DEP3	0.14*	0.25***	0.32***	0.23***	0.35***	0.43***	0.49***	0.68***	-						
mDEP1	0.18***	0.26***	0.20**	0.39***	0.26***	0.28***	0.99***	0.62***	0.48***	-					
mDEP2	0.12	0.25***	0.23***	0.24***	0.39***	0.34***	0.63***	0.99***	0.66***	0.63***	-				
mDEP3	0.13*	0.23**	0.29***	0.21***	0.34***	0.39***	0.49***	0.67***	0.99***	0.49***	0.66***	-			
RU1	0.06	0.21**	0.09	0.23***	0.14*	0.15**	0.68***	0.49***	0.34***	0.68***	0.50***	0.35***	-		
RU2	− 0.004	0.19**	0.06	0.13*	0.18**	0.13*	0.36***	0.58***	0.45***	0.36***	0.57***	0.44***	0.56***	-	
RU3	− 0.02	0.12	0.12	0.10	0.18**	0.21***	0.34***	0.47***	0.67***	0.34***	0.45***	0.66***	0.40***	0.57***	-
**Full Sample**															
*M*	12.94	13.23	13.16	22.09	23.21	23.56	8.12	7.73	7.77	7.20	6.75	6.74	11.26	10.19	10.43
*SD*	3.90	4.00	4.64	8.32	8.79	9.02	7.16	7.28	7.13	6.53	6.61	6.38	8.77	8.66	9.03
*n*	345	223	251	352	301	308	356	299	308	356	299	306	357	301	310
**3rd Grade**															
*M*	12.95	12.76	13.13	19.15	21.12	21.85	6.85	6.40	6.34	5.99	5.60	5.51	10.63	7.92	7.68
*SD*	3.80	3.31	4.63	7.05	8.86	9.07	5.72	7.07	6.63	5.31	6.42	6.01	7.12	6.91	7.29
*n*	112	71	91	110	99	106	112	99	106	112	99	105	112	99	107
**6th Grade**															
*M*	12.72	13.54	12.81	22.25	23.27	23.87	7.04	7.44	8.28	6.26	6.56	7.23	9.67	9.56	11.12
*SD*	4.19	4.35	4.57	8.72	8.64	9.36	7.20	7.38	7.73	6.48	6.70	6.87	8.78	8.62	9.73
*n*	124	81	88	125	108	108	125	106	108	125	106	108	126	108	109
**9th Grade**															
*M*	13.17	13.35	13.64	24.68	25.34	25.14	10.46	9.46	8.80	9.32	8.18	7.56	13.55	13.30	12.77
*SD*	3.67	4.22	4.76	8.15	8.45	8.31	7.78	7.10	6.75	7.12	6.53	6.06	9.73	9.49	9.24
*n*	109	71	72	117	94	94	119	94	94	119	94	93	119	94	94

*IN* caregiver-reported insomnia, *SLP* child-reported sleep problems, *DEP* child-reported depression, *mDEP* child-reported depression, with sleep and tiredness items removed, *RU* child-reported rumination

Correlation matrix based on full sample. *p* < .001***, *p* < .01**, *p* < .05*

### Primary CLPM

The primary CLPM examined prospective relationships and mediational pathways among caregiver-reported insomnia, child-reported depression, and child-reported rumination. Fit indices were calculated for CLPMs with (1) unconstrained regressive paths (AIC = 16803, BIC = 16979, RMSEA = 0.079), (2) constrained autoregressive paths (AIC = 16800, BIC = 16964, RMSEA = 0.068), and (3) constrained autoregressive and cross-lagged paths (AIC = 16801, BIC = 16941, RMSEA = 0.065). The fully constrained model (i.e., with both autoregressive and cross-lagged paths constrained to be equal across time) exhibited the lowest BIC and RMSEA, with AIC just one point higher than the model with autoregressive constraints alone. Based on this comparison, the fully constrained model was selected for further analysis. Next, the covariates of sex (0 male, 1 female) and grade (3, 6, 9) were included in the model, with effects on each observed variable constrained to be equal at each time point. Model fit for the final CLPM was excellent, with fit indices meeting recommended thresholds (Hu & Bentler, 1999): *χ²*(30) = 64.79, *p* < .001, CFI = 0.972, TLI = 0.950, and RMSEA = 0.056.

**Fig. 1:**
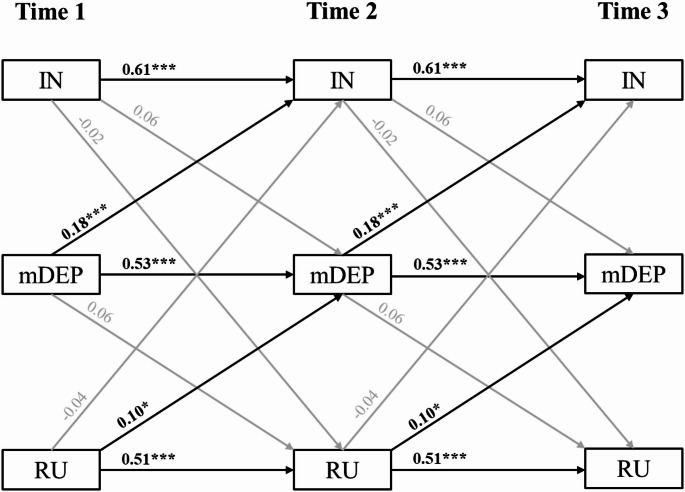
Primary cross-lagged panel model. Note: IN = caregiver-reported insomnia; mDEP = child-reported depression, with sleep and tiredness items removed; RU = child-reported rumination. Covariates included in models are not depicted. Standardized estimates reported. *p* < .001***, *p *< .01**, *p* < .05*

CLPM results are presented in Table 2 and depicted in Fig. 1. Standardized estimates (β) are reported in the text. Of note, standardized estimates vary slightly across time points due to differences in variances, despite equality constraints on unstandardized paths. Insomnia (β = 0.61), depression (β = 0.53), and rumination (β = 0.51) all demonstrated significant autoregressive effects (all *p*s < .001). Depression significantly predicted greater subsequent insomnia over time, with a large effect size (β = 0.18, *p* < .001). Meanwhile, the cross-lagged effect of insomnia on depression only reached trend-level significance (β = 0.06, *p* =.072). Rumination significantly predicted greater subsequent depression over time, with a medium effect size (β = 0.10, *p* = .014). Though, this relationship was not reciprocal, as depression did not predict later rumination (*p* = .427). Cross-lagged pathways between rumination and insomnia were also non-significant (*p*s = .427- .539). Being in a higher grade and female was associated with higher levels of both depression and rumination, but grade and sex were not significantly related to insomnia.

**Table 2:** Primary cross-lagged panel model results

Path	β	*b*	*SE*	*p*	95% Bootstrapped CI	
*Autoregressive paths (constrained)*						
IN_T_ → IN_T+1_	0.61	0.64	0.04	**< 0.001**	0.53	0.74
mDEP_T_ → mDEP_T+1_	0.53	0.54	0.04	**< 0.001**	0.43	0.65
RU_T_ → RU_T+1_	0.51	0.50	0.04	**< 0.001**	0.39	0.60
*Cross-lagged paths (constrained)*						
IN_T_ → mDEP_T+1_	0.06	0.10	0.06	0.072	−0.03	0.23
mDEP_T_ → IN_T+1_	0.18	0.11	0.03	**< 0.001**	0.04	0.19
IN_T_ → RU_T+1_	−0.02	−0.05	0.08	0.539	−0.19	0.12
RU_T_ → IN_T+1_	−0.04	−0.02	0.02	0.427	−0.06	0.03
mDEP_T_ → RU_T+1_	0.06	0.09	0.06	0.153	−0.05	0.23
RU_T_ → mDEP_T+1_	0.10	0.07	0.03	**0.014**	0.01	0.15
*Covariates (constrained)*						
female → IN	−0.02	−0.16	0.24	0.503	−0.64	0.31
grade → IN	0.01	0.01	0.49	0.842	−0.09	0.11
female → mDEP	0.09	1.18	0.34	**< 0.001**	0.50	1.87
grade → mDEP	0.07	0.20	0.07	**0.006**	0.06	0.34
female → RU	0.08	1.32	0.48	**0.006**	0.37	2.28
grade → RU	0.12	0.43	0.10	**< 0.001**	0.25	0.63
*Indirect effects*						
(IN_T_ → mDEP_T+1_) x (mDEP_T+1_ → RU_T+2_)	0.00	0.01	0.01	0.241	−0.01	0.03
(IN_T_ → RU_T+1_) x (RU_T+1_ → mDEP_T+2_)	0.00	0.00	0.01	0.548	−0.02	0.01
(mDEP_T_ → IN_T+1_) x (IN_T+1_ → RU_T+2_)	0.00	−0.01	0.01	0.547	−0.02	0.02
(mDEP_T_ → RU_T+1_) x (RU_T+1_ → IN_T+2_)	0.00	0.00	0.00	0.501	−0.01	0.00
(RU_T_ → IN_T+1_) x (IN_T+1_ → mDEP_T+2_)	0.00	0.00	0.00	0.470	−0.01	0.00
(RU_T_ → mDEP_T+1_) x (mDEP_T+1_ → IN_T+2_)	0.02	0.01	0.00	**0.038**	0.00	0.02

*IN* caregiver-reported insomnia, *mDEP* child-reported depression, with sleep and tiredness items removed, *RU* child-reported rumination

Confidence intervals (CI) were obtained from 5,000 bootstrapped samples

Standard errors (*SE*) and *p*-values are based on standard estimation. Standardized estimates (β) vary slightly across time points due to differences in variances, despite equality constraints on unstandardized paths. Standardized estimates for the initial time point are reported

Of the six possible sequences tested, only the indirect effect of rumination (predictor) on insomnia (outcome) through depression (mediator) emerged as significant (β = 0.02, *p* =.038). As reflected in the cross-lagged effects, greater rumination predicted greater depression, which in turn predicted greater insomnia. This indirect 

### Secondary CLPM

This analytic approach was replicated in a second CLPM, substituting child-reported sleep difficulties for the caregiver-reported insomnia measure. Model fit indices were mixed: the fully constrained model showed the lowest AIC and BIC values (AIC = 18974, BIC = 19114, RMSEA = 0.042), while the model with only autoregressive constraints had the lowest RMSEA (AIC = 18976, BIC = 19139, RMSEA = 0.018). The unconstrained model had the poorest fit (AIC = 18979, BIC = 19154, RMSEA = 0.078). The fully constrained model was selected for further analysis due to its greater degrees of freedom and consistency with the primary model. With covariates included, the final model demonstrated good fit: *χ²*(30) = 69.34, *p* < .001, CFI = 0.970, TLI = 0.946, and RMSEA = 0.060.

**Fig. 2:**
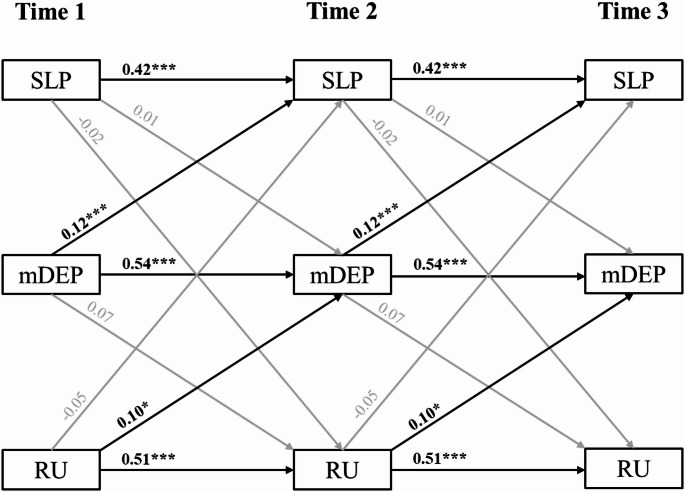
Secondary cross-lagged panel model. Note: SLP = child-reported sleep problems; mDEP = child-reported depression, with sleep and tiredness items removed; RU = child-reported rumination. Covariates included in models are not depicted. Standardized estimates reported. *p* < .001***, *p *< .01**, *p* < .05*

Results for the secondary model are presented in Table 3 and depicted in Fig. 2. In this model, depression significantly predicted later sleep difficulties with a large effect size (β = 0.12, *p* = .008), but sleep difficulties did not predict later depression (*p* = .846). Consistent with the primary model, rumination significantly predicted subsequent depression over time, with a medium-sized effect (β = 0.09, *p* = .015). Unlike in the primary model which utilized a caregiver-report measure of insomnia, covariates were associated with youth-reported sleep difficulties. Being in a higher grade was significantly associated with greater sleep difficulties, and female sex was associated with greater sleep difficulties at the significance threshold (*p* = .050).

**Table 3:** Secondary cross-lagged panel model results

Path	β	*b*	*SE*	*p*	95% Bootstrapped CI	
*Autoregressive paths (constrained)*						
SLP_T_ → SLP_T+1_	0.42	0.47	0.04	**< 0.001**	0.36	0.57
mDEP_T_ → mDEP_T+1_	0.54	0.55	0.04	**< 0.001**	0.43	0.65
RU_T_ → RU_T+1_	0.51	0.51	0.04	**< 0.001**	0.40	0.61
*Cross-lagged paths (constrained)*						
SLP_T_ → mDEP_T+1_	0.01	0.01	0.03	0.846	−0.04	0.05
mDEP_T_ → SLP_T+1_	0.12	0.18	0.07	**0.008**	0.00	0.34
SLP_T_ → RU_T+1_	−0.02	−0.02	0.04	0.529	−0.10	0.05
RU_T_ → SLP_T+1_	−0.05	−0.06	0.05	0.226	−0.17	0.06
mDEP_T_ → RU_T+1_	0.07	0.09	0.06	0.146	−0.05	0.24
RU_T_ → mDEP_T+1_	0.10	0.07	0.03	**0.015**	0.01	0.15
*Covariates (constrained)*						
female → SLP	0.06	0.97	0.49	0.051	−0.07	2.05
grade → SLP	0.12	0.42	0.11	**< 0.001**	0.21	0.65
female → mDEP	0.09	1.16	0.34	**0.001**	0.47	1.85
grade → mDEP	0.07	0.19	0.07	**0.008**	0.06	0.34
female → RU	0.08	1.34	0.49	**0.006**	0.37	2.30
grade → RU	0.12	0.44	0.10	**< 0.001**	0.25	0.63
*Indirect effects*						
(SLP_T_ → mDEP_T+1_) x (mDEP_T+1_ → RU_T+2_)	0.00	0.00	0.00	0.844	−0.01	0.03
(SLP_T_ → RU_T+1_) x (RU_T+1_ → mDEP_T+2_)	0.00	0.00	0.00	0.539	−0.01	0.01
(mDEP_T_ → SLP_T+1_) x (SLP_T+1_ → RU_T+2_)	0.00	0.00	0.01	0.544	−0.02	0.02
(mDEP_T_ → RU_T+1_) x (RU_T+1_ → SLP_T+2_)	0.00	−0.01	0.01	0.373	−0.03	0.00
(RU_T_ → SLP_T+1_) x (SLP_T+1_ → mDEP_T+2_)	0.00	0.00	0.00	0.847	0.00	0.00
(RU_T_ → mDEP_T+1_) x (mDEP_T+1_ → SLP_T+2_)	0.01	0.01	0.01	0.051	−0.001	0.02

*SLP* child-reported sleep problems, *mDEP* child-reported depression, with sleep and tiredness items removed, *RU * child-reported rumination

Confidence intervals (CI) were obtained from 5,000 bootstrapped samples

Standardized estimates (β) vary slightly across time points due to differences in variances, despite equality constraints on unstandardized paths. Standard errors (*SE*) and *p*-values are based on standard estimation. Standardized estimates for the initial time point are reported

All six mediational pathways were modelled. The indirect effect of rumination (predictor) on sleep difficulties (outcome) through depression (mediator) exhibited trend-level significance (β = 0.01, *p* =.051). No other mediational pathways were significant. This indirect effect was not significant when tested using 5,000 bootstrapped samples (95% CI [-0.001, 0.03]).

## Discussion

The present study investigated reciprocal relationships among insomnia, depression, and rumination, examining their developmental pathways over three time points (baseline, 1 year, and 2 years) in a community sample of youth. On average, participants experienced elevated, but subclinical, difficulties initiating and maintaining sleep (Bruni et al., 1996) and nonclinical levels of depression (Reynolds, 1998). We hypothesized that insomnia and depression would exhibit a bidirectional relationship, with rumination serving as a mediator in the development of these conditions. Instead, CLPM findings revealed a distinct pattern in which rumination predicted later depression, and depression predicted later insomnia. Notably, insomnia predicted later depression only at trend-level significance and was not associated with rumination. These results reflect relationships when controlling for effects of grade cohort and sex on observed variables at each time point. Contrary to hypothesis, rumination did not mediate prospective associations between depression and insomnia. One mediational pathway emerged demonstrating that early rumination contributed to later insomnia through depression. This developmental sequence was further supported by a secondary CLPM that replaced caregiver-reported insomnia with a measure of child-reported sleep/wake difficulties, yielding a consistent pattern of results- albeit at trend-level significance (*p* = .051)- when all variables (i.e., depression, rumination, and sleep) were based on youth self-report.

###  Relationship Between Insomnia and Depression

Given few studies have examined bidirectional relationships among sleep disturbances and depression in youth, one of the primary aims of this study was to examine prospective relationships across insomnia and depressive symptoms. Cross-sectionally, youth-rated depression was correlated weakly with caregiver-reported insomnia and moderately with youth-reported sleep/wake difficulties. The primary CLPM testing cross-lagged relationships found evidence that greater depression predicted greater next year insomnia over time, with consistent findings for youth-reported sleep. However, limited evidence emerged for a reciprocal relationship, as insomnia only predicted next year depression at trend-level significance (*p *= .072). The absence of a significant cross-lagged effect of insomnia on depression was not due to the use of the caregiver-reported measure, as youth-reported sleep difficulties failed to predict depression (*p *= .846). Importantly, significant findings were not attributable to construct overlap, as analyses utilized a modified depression variable with sleep and tiredness items removed. 

The finding that depression predicted insomnia- while evidence for the reverse pathway was weak-to-absent- is notable given sleep has been thought to be a robust risk factor for depression (Franzen & Buysse, 2008). Indeed, before evidence emerged supporting bidirectionality among youth (Marino et al., 2022; Sivertsen et al., 2021), most research suggested the relationship was unidimensional, with sleep disturbances predicting the onset of depression in children and adolescents (Alvaro et al., 2013; Lovato & Gradisar, 2014). That said, other studies have failed to find consistent bidirectional sleep-depression relationships among youth (Marino et al., 2022; Quach et al., 2018; Richardson et al., 2025), and studies with adults have found the same pattern of results as the present study using random intercept and traditional cross-lagged panel modeling (Tsui & Chan, 2024; Zhou et al., 2024). While conclusions from the body of literature supports early insomnia symptoms as a risk factor for later depression, results of the present investigation highlight an alternative developmental pathway for these conditions.

### Developmental Pathways

This study identified a mediational pathway whereby depression mediated the effect of rumination on insomnia. This indirect effect was modelled in the CLPM as the cross-lagged effect of rumination on depression (path a) by the cross-lagged effect of depression on insomnia (path b). Despite constraints on cross-lagged paths to be equal over time, this approach is still considered a test of longitudinal mediation as the model retains temporal precedence of the predictor (baseline), mediator (year 1), and outcome (year 2) variables. This mediational pathway was significant using standard estimation in the primary CLPM and was replicated in the secondary model using a youth-reported sleep variable with a *p*-value of 0.051. While the indirect effect was no longer significant when estimated using 5,000 bootstrapped samples, cross-lagged paths from rumination to depression and depression to insomnia remained significant with bootstrapping, reinforcing support for this developmental sequence. This study failed to identify support for any of the other five mediation sequences tested, including the hypothesized pathway whereby rumination would mediate prospective relationships between insomnia and depression.

The role of rumination in the sleep and depression link remains under-investigated, particularly in youth, and research across the lifespan has been limited by designs which do not clarify temporal relationships among these conditions. Meanwhile, a recent naturalistic study with adolescents (beginning at age 10–12) stands out for its analytic strategy testing several developmental pathways over 6 annual assessments (Richardson et al., 2025). These researchers found the most support for a mediation pathway where rumination mediated the effect of sleep disturbance on depression over time. There was also support for sleep disturbances mediating the effect of rumination on depression over time. However, the mediational pathway identified in the present study was not supported by Richardson and colleagues, potentially due to the measurement of sleep.^1^ Given evidence for alternative mediational pathways across sleep, depression, and rumination in the literature (Li et al., 2024; Richardson et al., 2025; Slavish & Graham-Engeland, 2015; Tsui & Chan, 2024), it may be that there are several developmental sequences for these conditions. More research is needed to determine whether variability in sample characteristics, including developmental stage, and design characteristics, such as measurement of sleep and rumination, may account for some of the exhibited differences.

Regarding characteristics of the present sample, this is the first study to our knowledge to examine these relationships across childhood. The CLPMs included grade cohort and sex as covariates, finding that higher grade was associated with greater severity of depression, rumination, and youth-reported (but not caregiver-reported) sleep problems. Female participants also exhibited higher severity, particularly for depression and rumination, in line with prior research (Hilt et al., 2010; Nolen-Hoeksema et al., 2008; Watkins & Roberts, 2020). Importantly, results lend support to the view that depressive rumination contributes to the development of mood and insomnia during youth, controlling for the impact of grade and sex on the severity of these variables. However, limited sample sizes precluded a thorough examination of whether the strength or direction of cross-lagged effects varied as a function of developmental stage or sex. Future research with larger samples should utilize moderation or subgroup analyses to test age and sex-related differences.

### Rumination

While it is expected that several biological and environmental factors underpin the sleep and depression link, identification of modifiable behavioral mechanisms is of particular clinical salience for the purposes of prevention and intervention (Blake et al., 2018). Previous studies identified that rumination may be one such mechanism linking sleep problems and depression (Li et al., 2024; Richardson et al., 2025; Slavish & Graham-Engeland, 2015; Tsui & Chan, 2024; Wang et al., 2023), though more research is needed to understand the role repetitive negative thinking plays in the development of depression and sleep conditions. The present study focused on rumination which occurs in the context of low mood, often referred to as depressive rumination.

Cross-sectionally, this measure was strongly correlated with depression but was weakly or not significantly correlated with sleep variables. In the CLPM, rumination predicted subsequent depression, but depression did not predict later rumination. These results align with long-standing findings that rumination is a robust risk factor for depression onset (Hilt et al., 2010; Nolen-Hoeksema et al., 2008; Watkins & Roberts, 2020). While predictive of depression, this study suggests depressive rumination does not directly affect the development of insomnia. Rumination was unrelated to insomnia in the CLPM (i.e., no significant cross-lagged relationships). Instead, effects for rumination on insomnia appear to be fully mediated by depression. However, these results do not rule out the possibility other types of repetitive negative thinking (e.g., worry) or forms of rumination (e.g., rumination occurring before sleep onset, rumination specifically about sleep problems) may more directly contribute to the development of insomnia in youth. Unhelpful beliefs about sleep and perseverative cognition occurring before bed are central to cognitive theories of insomnia (Tang et al., 2023). For these reasons, it is important that the present study’s findings be interpreted within the context of general, trait-like tendencies toward depressive rumination.

###  Limitations

We would like to acknowledge limitations of this work. First, to maximize statistical power, analyses were conducted using the full sample, aggregating participants across grades to demonstrate overall associations in this foundational assessment. Results should be interpreted with caution regarding developmental implications, as future research with larger samples is needed to clarify nuanced age-related differences. Second, while the indirect effect of rumination on insomnia through depression was significant using standard estimation, it was no longer significant when estimated with 5,000 bootstrapped samples- suggesting this effect may be sensitive to sampling variability or that the model may potentially be underpowered. Importantly, both path a (rumination to depression) and path b (depression to insomnia) remained significant in the bootstrapped model, lending support to this developmental sequence; however, replication is needed to more confidently establish this mediation pathway.

Third, while this study advances our understanding of depressive rumination in relation to depression and insomnia, findings may not generalize to other ruminative subtypes, such as sleep-related or content-independent rumination. Further research is also needed to determine if results for depression extend to other related and clinically important constructs, such as stress and anxiety, which are known to be associated with sleep and rumination (e.g., Alloy et al., 2012; Brown et al., 2018). Fourth, the sample exhibited average, nonclinical depression severity and elevated but subclinical insomnia severity. These relationships may play out differently in those with more severe symptoms. The present findings also leverage widely used, though older measures. While the focus was on variable relationships, future research should incorporate the most recent measures to reference current norms. Finally, while not necessarily a limitation, assessments occurred over 1-year periods. This study and others have successfully identified relationships across 1-year timeframes and longer (Marino et al., 2022; Richardson et al., 2025; Sivertsen et al., 2021). Still, more research is needed examining varying time windows to capture the development of sleep and mood conditions across youth.

## Conclusion

Sleep and mood disturbances that begin early in life are linked to greater severity and chronicity over time, highlighting the need to understand developmental processes and modifiable factors that contribute to the onset of insomnia and depression in youth. This investigation contributes evidence for a developmental sequence in which depressive rumination contributes to later depression which in turn contributes to later insomnia among youth. Future research is needed to further clarify age-specific trajectories from childhood to adolescence. These results may inform early intervention and prevention efforts, as they suggest targeting depressive rumination may help mitigate the risk of developing depression and insomnia. Future research should examine the impact of intervening on tendencies to ruminate in response to low mood early in development. 

## Data Availability

The data presented in this study may be made available upon reasonable request to corresponding author and principal investigator, Dr. Carla Kmett Danielson.

## Footnotes

1 In Richardson et al. (2025), depression did not consistently predict worse sleep in their models (path b). Instead, depression either showed no predictive effect or predicted *better* sleep outcomes. These unexpected results may stem from sleep being measured as a latent factor, with sleep duration heavily weighted. Given depression is also related to hypersomnolence (i.e., longer sleep durations which corresponded to “better” sleep), this may have obscured the relationship between depression and sleep in their study.
